# Spiritual care in the outpatient environment for chronically ill older African American patients: Protocol for a pilot feasibility study

**DOI:** 10.1371/journal.pone.0337436

**Published:** 2025-11-24

**Authors:** Melinda Xu, Adrianne Smiley, Anita Aboagye, Tamara Nix Parker, Kameron Phillips, Kwaku Duah Oppong, Alexia M. Torke, Shelley Varner Perez, Sarah Khalidi, George Fitchett, Raegan W. Durant, Deborah Ejem

**Affiliations:** 1 School of Health Professions, University of Alabama at Birmingham, Birmingham, Alabama, United States of America; 2 Department of Occupational Therapy, School of Health and Rehabilitation Sciences, University of Pittsburgh, Pittsburgh, Pennsylvania, United States of America; 3 School of Education and Health Sciences, University of Alabama at Birmingham, Birmingham, Alabama, United States of America; 4 School of Nursing, University of Alabama at Birmingham, Birmingham, Alabama, United States of America; 5 Department of Political Science and Public Administration, University of Alabama at Birmingham, Birmingham, Alabama, United States of America; 6 School of Nursing, University of Texas at Austin, Austin, Texas, United States of America; 7 Indiana University Center for Aging Research, Regenstreif Institute Inc., Indianapolis, Indiana, United States of America; 8 Evans Center for Spiritual and Religious Values in Healthcare, Indiana University Health, Indianapolis, Indiana, United States of America; 9 Department of Religion, Health and Human Values, Rush University Medical Center, Chicago, Illinois, United States of America; 10 Heersink School of Medicine, University of Alabama at Birmingham, Birmingham, Alabama, United States of America; PLOS: Public Library of Science, UNITED KINGDOM OF GREAT BRITAIN AND NORTHERN IRELAND

## Abstract

**Background:**

Spirituality is vital to holistic patient care and should be proactively addressed by healthcare providers as unmet spiritual needs are a major source of suffering for patients living with chronic illnesses. However, spiritual concerns are seldom referenced by clinicians during disease-related treatment discussions, particularly for minority and under-resourced patients. This paper outlines a protocol for administering and evaluating a culturally-responsive spiritual care intervention conducted with chronically ill African Americans receiving care at a community safety net health service.

**Methods:**

A total of 60 African American patients who have chronic conditions and are aged 50 or older will be recruited for this study and randomly assigned to either the intervention (spiritual care program delivered by a board-certified chaplain) or the control group. All participants will complete a baseline interview that encompasses demographic information, religiosity, spiritual well-being, quality of life, and perception of care. Follow-up surveys will be conducted 12 weeks post-baseline, and participants assigned to the intervention group will also undergo a semi-structured acceptability and feasibility interview. Effect size measures and bivariate tests will be used to compare pre- and post-test outcomes while the interviews will be analyzed using constant comparative and thematic analysis.

**Discussion:**

Study findings will assess the feasibility and effectiveness of a culturally-responsive spiritual care intervention for under-resourced African American patients with chronic illnesses. Furthermore, the provision of spiritual care may help patients clarify their healthcare values and decision making priorities.

## Introduction

Chronic diseases are persistent health conditions that require ongoing medical attention and may interfere with daily functioning. Such illnesses are also the leading cause of disease, disability, and death in the United States (US), and certain populations are at greater risk of developing chronic diseases [1]. African American (AA) Medicare beneficiaries have higher prevalence rates of chronic conditions such as hypertension, diabetes, and stroke in comparison to their White counterparts, with over a third of AA beneficiaries reporting fair or poor health [2].

Palliative care (PC) can mitigate the burden of chronic illness by enhancing quality of life and addressing the emotional, physical, and spiritual distress experienced by patients and their families. Although it can be introduced at any point during the course of a serious illness and continue throughout the disease progression to end-of-life (EOL) [3], PC and EOL care are underutilized among AA patients despite their high burden of chronic disease [4,5]. Barriers that contribute to these disparities in usage include a lack of information and poor perceptions of EOL care, incongruence with cultural values, and mistrust of the medical system [6–10]. In addition, this underuse may also arise from the absence of culturally-responsive communication that would prompt AA patients with chronic illnesses to identify their values and priorities [6,11].

For many individuals, spirituality is an essential component of the human experience and must be proactively addressed by healthcare providers to deliver care that honors the whole person [12]. For patients with life-limiting illnesses, spirituality can offer vital support in coping with their condition with many considering it fundamental to the healing process [12,13]; some individuals may make critical healthcare decisions on the basis of their religious or spiritual beliefs [14]. Among older AA adults with chronic conditions, prayer and faith have been expressed as important coping mechanisms [15]. Yet, spiritual considerations are seldom referenced by clinicians during disease-related treatment discussions [16], especially for patients from minority and under-resourced populations [17].

Recognizing the importance of these concerns, the 4^th^ Edition of the Clinical Practice Guidelines for Quality Palliative Care identifies spiritual, religious, and existential aspects of care as one of the key domains integral to delivering high-quality PC [18]. Spiritual care (SC) aims to recognize and respond to the needs of patients as they cope with trauma, illness, or grief [19]. Addressing spiritual needs is particularly important for addressing the structural inequalities related to PC and EOL care. AAs, for example, frequently experience poor communication regarding care options and goals of care [20], highlighting the necessity for culturally-responsive conversations and education regarding EOL decision making [6]. Ongoing spiritual support could help patients and family members be more accepting of PC and EOL care options [8].

This paper describes the study protocol for a culturally adapted SC intervention designed to support older AAs with heart failure (HF), chronic kidney disease (CKD), or chronic obstructive pulmonary disease (COPD) who are receiving care at a community safety net health service. This study aims to assess the acceptability, feasibility, and preliminary impact of the intervention on spiritual well-being, quality of life, and perception of care. HF, CKD, and COPD represent a substantial share of the chronic disease burden among AAs [21]; yet AAs living with these conditions remain underrepresented in clinical trials [22–24]. To address this gap, our study focuses on this population to generate data that can inform person-centered, culturally-responsive care. By centering the lived experiences and needs of older AAs with chronic illness, we aim to support the development of evidence-based practices that reduce disparities and improve outcomes in a group often overlooked in clinical research.

## Methods

### Theoretical model

The study is informed by the Biopsychosocial-Spiritual Model of Care (BSMC) which supports a holistic view of the patient and recognizes spirituality (i.e., an individual’s search for transcendent meaning) as a fundamental aspect of human well-being [25]. The model posits that spiritual well-being influences not only health behaviors but also how patients experience and cope with illness. Serious illness can disrupt spiritual connectedness, leading to distress that may affect relationships with the self, others, and the transcendent. SC interventions grounded in the BSMC aim to restore balance across biological, psychological, and spiritual domains—especially for chronically ill patients in under-resourced communities.

### Study design, setting, and sample

The study will use a randomized multi-method approach. Participants will be recruited from the cardiac, pulmonary, and renal clinics of Cooper Green Mercy Health Services Authority (CGMHSA), a publicly funded, safety net health system located in Jefferson County, Alabama.

Eligible participants will: (1) self-identify as AA, (2) be at least 50 or older, (3) speak English, (4) have a diagnosis of a least one of the following conditions based on chart review—HF, CKD, or COPD, 5) be cognitively able to participate in the SC intervention and complete study questionnaires, and 6) have reliable telephone access. Patients will be excluded if they: (1) have an Axis I psychiatric (e.g., schizophrenia, bipolar disorder) disorder, dementia, or active substance use disorder or (2) reside in a nursing home or assisted living facility.

### Recruitment procedures

Recruitment will utilize previously successful procedures [26] in which the eligibility criteria (outlined above) are applied as screening parameters to identify patients through an electronicmedical record (EMR) review of diagnostic codes; a comprehensive list of patients meeting those criteria was generated and securely transmitted to the Principal Investigator via an encrypted electronic file. Eligible participants will receive an opt-out letter and a flyer by mail, detailing the study’s purpose and procedures. The letter will inform recipients that they will be contacted in two weeks unless they call the study phone number to opt out. After the two-week opt-out window, study staff will contact individuals who have not declined participation to provide details about the study. During this call, study staff will assess the individual’s interest in participating in the SC program. Participants expressing interest will be consented through Institutional Review Board (IRB)-approved verbal consent. Patients will receive $25 for each data collection activity they complete—baseline questionnaire, 12-week post-baseline follow-up questionnaire, and semi-structured interview (intervention group only)—for a maximum of $75 for completion of all study activities for intervention group participants and $50 for usual care group participants.

### Randomization

**Fig 1:**
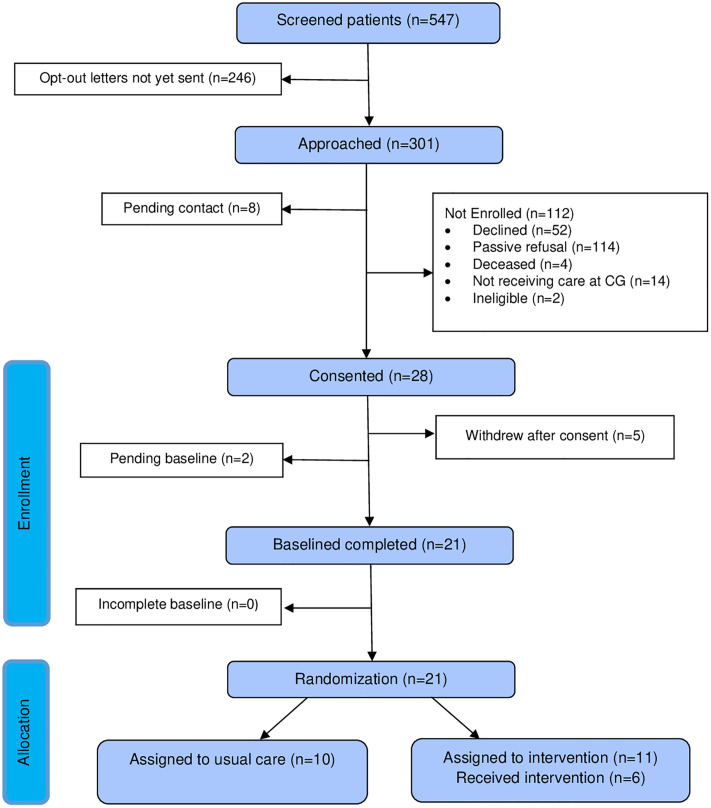
Flowchart of study procedures.

Following the completion of the baseline interview, participants will be randomly assigned to receive either usual care from CGMHSA or the culturally-responsive SC intervention administered by a trained SC specialist (i.e., a board-certified chaplain) using stratified block randomization by sex and clinic type in REDCap. Since the intervention is administered as a feasibility pilot, the Principal Investigator and study staff will not be blinded. The participant flow is depicted in Fig 1.

### Spiritual Care and Assessment Intervention (SCAI)

The SCAI is a chaplain-delivered SC intervention comprised of four core components: (1) proactive contact, (2) a semi-structured spiritual assessment, (3) individualized SC, and (4) documentation of interaction [27]. SCAI is structured around four dimensions: Meaning and Purpose, Relationships, Transcendence and Peace, and Self-worth and Identity [27]. Originally designed for family members of seriously ill patients, SCAI has demonstrated feasibility and acceptability among advanced-stage cancer outpatients [28] and improved the spiritual and psychological outcomes for surrogate decision makers in intensive care settings [29]. However, this intervention has not yet been evaluated for acceptability or feasibility among AA patients. Previous qualitative interviews with AA patients and clinicians suggested adaptations to increase cultural responsiveness, including reducing the intervention length from 90 down to 60 minutes and delivering it in person [30]. These modifications will be incorporated in the current pilot.

Patients assigned to receive the SCAI will be contacted by an SC specialist to schedule an in-person appointment that will take place in-clinic at the participant’s convenience. If the participant is unable to meet in person for any reason, the session will occur instead over Zoom. At the visit, the SC specialist will ask semi-structured questions pertaining to each of the SCAI’s dimensions to gauge the patient’s spiritual needs. During this assessment, patients may be asked about their sources of strength, their connections to other people, and any stress they may be experiencing [27]. The SC specialist will then tailor and administer the intervention according to their perception of the participant’s specific needs; potential interventions may include active listening, prayer, reading of sacred texts, or emotional support [27]. Patients will also receive one follow-up phone call after the initial visit. At this time, the participant and SC specialist may reflect on, revisit, or explore their previous discussions in greater depth.

To ensure intervention fidelity, all SC specialists are required to achieve at least 80% adherence to the SCAI protocol, as assessed through a structured fidelity checklist. The SC specialists will undergo a standardized four-hour training delivered by the original developers of the intervention and will participate in ongoing quarterly fidelity monitoring and booster training sessions conducted by certified SCAI interventionists throughout the recruitment and implementation period. These sessions will reinforce core components of the intervention, address implementation challenges, and promote consistency in delivery across sites and time points.

### Study procedures

60 participants will complete pre-test measures at baseline, after which half will be randomized to receive usual care, in addition to post-test measures following the completion of the intervention. Questionnaires will include demographic information, spiritual well-being (Functional Assessment of Chronic Illness Therapy – Spiritual Well-Being 12 Item Scale) [31], religiosity (Duke University Religion Index) [32], religious coping (Scale of Religious and Spiritual Coping) [33], quality of life (Functional Assessment of Cancer Therapy- General) [34], perception of care (Older Patient Assessment of Chronic Illness Care) [35], treatment burden (Treatment Burden Questionnaire) [36], distress (Distress Thermometer) [37], hope (Trait Hope Scale, State Hope Scale) [38,39], anxiety and depression (Hospital Anxiety and Depression Scale) [40]. For the purposes of this study, feasibility will serve as the primary outcome, with the others as secondary to explore preliminary impacts to guide subsequent larger studies.

### Acceptability and feasibility

Semi-structured acceptability interviews will be conducted with patients in the intervention arm two weeks after completing the SCAI program with the SC specialist. These interviews, conducted by study staff, will explore patients’ perceptions of the program, its impact, and any suggestions for improvements.

We aim for a 75% completion rate of study activities, defined as the proportion of enrolled participants who complete all components of the study protocol. This benchmark is consistent with retention rates of similar longitudinal clinical studies and reflects a realistic threshold to ensure the acceptability, feasibility, and relevance of our culturally tailored intervention to the target population [41–43].

### Spiritual well-being and religiosity

The Functional Assessment of Chronic Illness – Spiritual Well-Being 12 Item Scale (FACIT-SP-12) evaluates the spiritual well-being of individuals with cancer or other chronic illnesses [31]. With good internal reliability (ɑ = 0.81–0.88) and high internal consistency (ɑ = 0.96), the questionnaire is a valid and reliable measure of well-being across a variety of religious traditions and among ethnically diverse samples. The Scale of Religious and Spiritual Coping (RCOPE) assesses religious coping in regard to major life stressors and was initially tested among college students who had experienced a negative life event as well as hospitalized older adults [33]. The scale was found to have high internal consistency, incremental validity, and reliability. Meanwhile, the Duke University Religion Index (DUREL) measures religiosity across three major dimensions: intrinsic religiosity, organizational religious activity, and non-organizational religious activity. Previously administered to medical inpatients as well as older adults in large-scale epidemiologic studies, the DUREL has high test-retest reliability, high internal consistency (ɑ = 0.78–0.86), and high convergent validity with other measures of religious involvement [32].

### Quality of life, perception of care, and treatment burden

The Functional Assessment of Cancer Therapy- General (FACT-G) measures the quality of life of patients undergoing cancer treatment across the four domains of physical, social/family, emotional, and functional well-being. Although originally developed for individuals with cancer, it has been administered to populations with other chronic illnesses including heart disease, COPD, renal disease, and HIV/AIDS [34,44]. The instrument indicates high internal consistency (ɑ = 0.89) and test-retest reliability [34]; it was further found to be a valid and reliable scale when tested with older cancer patients [45].

The Older Patient Assessment of Chronic Illness Care (O-PACIC) is a 10-item instrument that assesses the perception of primary care from the perspective of older patients who have been recently discharged following hospitalization [35]. The O-PACIC shows good validity and internal consistency, and its short length entails that it can be feasibly administered to frail and chronically ill patients.

The Treatment Burden Questionnaire (TBQ) gauges treatment burden, or the ‘work’ of being a patient with chronic conditions and their impact on quality of life. Respondents are asked to rate the extent to which they consider particular aspects of their treatment to be a big problem or not a problem [36]. Originally developed in French, an English translation of the TBQ was administered to patients with chronic conditions across several countries including the US, United Kingdom, Canada, and Australia. Internal consistency (ɑ = 0.70–0.95) and test-retest reliability were further deemed to be acceptable**.**

### Distress, hope, and anxiety and depression

The Distress Thermometer instructs patients to rate their level of distress on a scale; scores range from 0 or “no distress” to 10 for “extreme distress.” Clinically relevant distress is signified by scores ≥ 3, and the scale is accompanied by an associated problem list of 34 issues that may have troubled respondents throughout the past week. Problems are arranged into categories: practical problems, emotional problems, spiritual/religious concerns, and physical problems [37]. Tested in cancer outpatients, the Distress Thermometer indicates a satisfactory level of accuracy and was found to be comparable to other distress measures.

The Trait Hope Scale measures the hope of a respondent according to Snyder’s cognitive model of hope; this model is based on a sense of successful agency or goal-directed determination as well as the perception of pathways for meeting goals [38]. The scale has been administered to college students as well as patients receiving psychological treatment, showing both high internal consistency (ɑ = 0.74–0.84) and good test-retest reliability. On the other hand, the State Hope Scale assesses the goal-directed thinking of individuals in the present moment and is responsive to the events of respondents’ lives [39]. Tested in samples of college students, the scale was found to be a valid and internally consistent measure of goal-directed determination and planning.

The Hospital Anxiety and Depression Scale (HADS) measures states of anxiety and depression for patients in a general medical clinic setting [46]. A score of 11 or higher indicates an abnormal case while scores in the range of 8–10 denote borderline cases. The HADS has been validated in elderly samples [46] and shows satisfactory internal consistency and test-retest reliability [47].

### Data analysis

#### Quantitative analysis.

Data from pre- and post-intervention questionnaires will be analyzed with SPSS; measures of effect size and bivariate tests of associations will be conducted to compare pre- and post-test measures. Due to the small sample size, broad patterns of responses will be examined.

#### Qualitative analysis.

Patient interviews will be transcribed verbatim and entered in NVivo where an iterative constant comparative method [48] and content thematic analysis [49] will be used to systematically identify, categorize, and interpret patterns in the data [50]. An a priori codebook will be developed based on the study’s conceptual framework, interview guide, and existing literature on spirituality, patient-centered care, and cultural tailoring. This initial codebook will include both deductive codes (informed by existing theories and constructs) and be flexible to accommodate inductive codes that emerge during analysis [51].

Two trained coders will independently apply the codebook to an initial set of transcripts, after which coding discrepancies will be discussed and resolved through consensus, with updates to the codebook made as needed. Constant comparison will be used throughout to refine codes, identify deviant cases, and develop robust thematic categories [52]. Saturation will be assessed through ongoing team discussions to determine when no new themes are emerging [53].

### Ethical standards

This study was reviewed and approved by the Institutional Review Board (IRB) at The University of Alabama at Birmingham. The IRB approval number is IRB-300008321. Throughout the recruitment process, study staff will obtain verbal informed consent from each participant prior to participation. Participants will be informed of the study’s purpose, procedures, potential risks and benefits, and their right to withdraw at any time without penalty. Verbal consent procedures were approved by the IRB in recognition of the minimal risk nature of the study.

### Results

The study was funded in July 2024 with recruitment commencing on September 6, 2024 and data collection beginning on December 30, 2024. Screening and enrollment of CGMHSA patients is ongoing. As of October 2025, 21 participants have been enrolled and randomized. Data collection is projected to conclude by June 2026, followed by data cleaning and analysis. Preliminary data analysis will begin in July 2026, with results anticipated for dissemination in Fall 2026 through conference presentations and peer-reviewed publications.

## Discussion

### Summary and significance

This protocol outlines the design of a pilot randomized clinical trial that explores the acceptability and feasibility of a culturally tailored SC intervention for older AA adults. This intervention is novel in its approach to addressing spiritual needs in a southern urban community, a geographical region that is often underrepresented in trial studies. Findings from this randomized feasibility study may inform the refinement and tailoring of spiritually integrated, culturally responsive, and patient-centered models of care aimed at reducing disparities and advancing equitable health outcomes.

### Strengths and anticipated challenges

The feasibility of the SC intervention may be shaped by multiple dimensions of primary care service delivery. Though the SC intervention is valuable for its potential to address knowledge gaps about holistic, patient-centered service delivery in primary care, anticipated challenges remain. Workflow integration is a primary consideration, as primary care facilities may lack the structural capacity to incorporate SC screenings or conversations into already time-constrained visits without disrupting clinical efficiency. Staff training and infrastructure readiness also raise questions about streamlining clear referral pathways to chaplains or community clergy for intervention uptake and utilization, as well as ways to reflect the receipt of SC services in the EHR if documentation systems do not account for spiritual assessments and services. Reimbursement and sustainability concerns represent additional implementation concerns, as staffing levels and productivity demands may not align with the added responsibilities of delivering SC.

To address the potential limitations of implementing the intervention, we are conducting this feasibility study at a single care site. This approach enables us to generate preliminary data on how SC can be effectively integrated into routine workflows, while minimizing the burden on staff without disrupting visit efficiency. The single-site study approach also allows us to explore whether brief, pre-implementation training is needed to support providers in introducing and documenting SC conversations that may facilitate intervention uptake. In addition, this feasibility study will allow us to examine referral pathways and uptake patterns across care recipient characteristics to characterize the intervention’s acceptability, appropriateness, and practicality within routine care.

### Future directions

Future research should focus on efficacy testing to determine the intervention’s effectiveness in improving clinical outcomes and its ability to produce sustained effects over longer periods of time. Studies should also examine the durability of intervention effects in community and natural care environments in response to health status changes. Efficacy testing in future studies may also assess the potential for implementation scaling in acute, short-term, and long-term care settings. In addition, future work should explore setting-specific adaptations and the potential development of structured protocols that may standardize SC delivery, guide provider training, and ensure fidelity, while allowing flexibility to accommodate diverse cultural and faith values so that enhancements to traditional care remain both responsive and relevant.
